# How to Measure the Safety Cognition Capability of Urban Residents? An Assessment Framework Based on Cognitive Progression Theory

**DOI:** 10.3389/fpsyg.2022.707172

**Published:** 2022-04-12

**Authors:** Yachao Xiong, Changli Zhang, Hui Qi, Rui Zhang, Yanbo Zhang

**Affiliations:** ^1^School of Public Policy and Management, China University of Mining and Technology, Xuzhou, China; ^2^School of Management, North China Institute of Science and Technology, Langfang, China; ^3^School of Management Engineering and Business, Hebei University of Engineering, Handan, China

**Keywords:** urban residents, safety cognition capability, conceptual structure, scale development, qualitative analysis

## Abstract

The salience of social risks and the incidence of various crises in China have induced widespread concerns among urban residents. Encountering frequent risks places higher demands on the cognition of urban residents. The concept of safety cognition capability is defined within the context of urban residents' daily life, and measurement instruments are developed and tested to lay the foundation for grasping the current safety cognition capability of urban residents and conducting further research. In this study, the five-dimensional structure of urban residents' safety cognition capability (URSCC) was proposed by using the grounded theory method to sort out the interview transcript of interviews with 30 urban residents, and a 38-item URSCC scale was designed and used for surveys conducted in China. The results show that the scale can be used as a valid tool to measure the URSCC, and it can help city managers to better understand the safety needs of residents, as well as monitor the effectiveness of policy implementation.

## Introduction

The transition from an industrial to a modern society symbolizes the onset of the “risk society,” in which people live with both conventional risks and new man-made uncertainties (Beck, 1992). Cities appear to be the areas with a high incidence of these natural and man-made hazards (Joffe et al., 2013; Singh, 2015). The side effects of urban modernization directly trigger risks or evolve into potential hazards (Frumkin, 2002; Ewing et al., 2016). Urban areas are not only victims but also producers of risks (Hood, 2005). As the coevolution of a sharp urban sprawl and rapid social transition takes place, major cities in China, especially megacities, are facing a surge of social risks and crises, which pose great challenges to local governments (Jinhua, 2018). The city is shrouded in thick smog (Cheng et al., 2017), and the location of some controversial neighboring facilities (Yue et al., 2018) indicate that Chinese urban residents are living in a high environmental hazards context. The continued and rampant public health safety scandals, such as the Sanlu Milk Powder and Changchun Vaccine incidents, vividly show that the Chinese are facing health risks related to food and medical care (Song et al., 2018; Wang and Ding, 2019). The frequent seasonal floods occurring in large cities have severely damaged important infrastructure, such as electricity and transportation, impacting people's daily lives, which is considered one of the most serious natural hazards occurring in Chinese cities. Accidental injuries caused by frequent risks usually occur during driving, in the workplace, and in the home environment (Hazinski et al., 2005). A survey shows that in 97% of emergencies, the first witnesses and community workers arrive at the scene before the professional emergency team (Bogdanski et al., 1999). If the public has a certain understanding of the risk and can handle emergency situations correctly, precious time can be gained. Accordingly, it is imperative to understand the safety cognition of urban residents in the Chinese context and provide a basis for risk mitigation and regulation policies.

Some scholars have put forward the concept of safety cognition capability and explored its measurement dimensions (Eby and Molnar, 2012; Honghai and Xu, 2014). The view they proposed was that safety cognition capability refers to the individual's identification and response to hazards in various activities, emphasizing the consideration of capabilities related to experience, knowledge, individual decisions, and collective behaviors. It is undeniable that accurate judgment and effective responses to hazards are the core elements of safety cognition capability, but one's hazard coping capability cannot be completely equal to one's safety cognition capability. As has been pointed out by the iceberg theory, the classic theory of capability research, capability is not limited to the values of knowledge and skills above the surface. Motivation hidden deep below the surface is the key to distinguishing differences in individual capability (Yu-Jie, 2012). Urban residents' capabilities can be easily observed, e.g., their knowledge, experiences, and behaviors, which are explicit characteristics, but the elements hidden, such as safety values, are rooted in the hearts of residents. These motivations are indispensable for understanding, evaluating, and improving their safety cognition capability.

Cognitive psychology is about processing information (Solso et al., 2005). The model of human information processing stages consists of four stages: sensory processing, perception, response selection, and execution selection (Wickens, 1984). The ladder model further refines the four stages of cognition into eight stages: activation, observation, recognition, interpretation, evaluation, definition of the task, formation of a protocol, and execution (Rasmussen, 1986). The generalized cognitive model divides cognitive processes into three different levels, the skill level, the rule level, and the knowledge level, which are in sequence of increasing levels of cognition. The individuals' cognitive processes are often only on the skill level and rule level (Reason, 1990). In conclusion, cognition has process discontinuity and degree difference, and urban residents' safety cognition also has similar characteristics. Urban residents from different social backgrounds have different cognition of safety, which means they are at different cognitive stages. However, few studies have paid attention to the cognitive gap among different groups. Previous research has focused on how to foster standardized crisis response behaviors among the public. Some researchers have attempted to build a standardized operational procedure for crisis communication that is universally applicable to the public (Fediuk et al., 2010). Standardized policies are also considered to be effective in improving public attitudes and behaviors toward food safety (Ma et al., 2019). In the infrequent scenario of earthquake disasters, disaster risk management agencies should regularly educate the public to maintain belief in the salience of disasters and the importance of preparedness (Zaremohzzabieh et al., 2021). The reality, however, is that there are differences in the upper limits of the individuals' capability to cope with risk and the capabilities needed to deal with hazards across different types of social backgrounds. Due to the constraints of their knowledge, skills, and experience, standardized education and policies of risk and disaster management agencies are not effective in reducing the gap in safety cognition between different groups. An efficient approach is to identify the state of safety cognition capability of various groups and develop targeted measures. Therefore, constructing a stage-based assessment framework to evaluate the safety cognition capabilities of groups from different social backgrounds in different risk situations can target the identification of individuals with deficient capabilities and their cognitive shortcomings.

Based on the statements above, this study introduces safety cognition capability into the daily life of residents and focuses on the development and testing of the urban residents' safety cognition capability (URSCC) scale, specifically: (1) on the basis of existing studies and in-depth interviews, the measurement items of the URSCC scale were refined through qualitative analysis and a preliminary research questionnaire was formed; (2) data were collected through a pre-study, and the scale structure was validated to improve the scale; (3) using formal research data, an exploratory factor analysis and a validation factor analysis were conducted on the scale; (4) the reliability and validity of the URSCC scale were analyzed.

City managers can use the URSCC scale to systematically address the cognitive gaps of residents and formulate targeted policies. This study aims to provide a new perspective for the study of urban residents' safety cognition.

## The Current Research

Capability is a stable psychological quality that refers to the possibility of an individual achieving various goals (Robbins and Judge, 2013). Studies in the field of psychology, philosophy, and organizational behavior believe that the generation and development of capability must be linked to specific tasks in specific situations (Chien and Tsai, 2012; De Vos et al., 2015; Stephens et al., 2015). Therefore, capability can also be understood as a possibility to accomplish a specific task. The greater the possibility, the stronger the individual's capability. Once a specific task is executed, there are two possibilities, namely success or failure, and the individual's capability is reflected in the transition from inability to ability regarding the task (Chien and Tsai, 2012). Therefore, capability is generally positive (Cavell, 1990). The possibility of the transition from incapability to ability among different individuals varies, that is, there are differences in capabilities between individuals. This conversion encompasses the whole process from the generation of individual capability to the individual's continuous development. Therefore, whether a person can complete a specific task is not only affected by his own knowledge and experience but, more importantly, their perception and attitude toward the task; that is, the value assigned to the task (McClelland, 1973). Values refer to the inherent evaluation of things, the overall view on ideas, customs, and social culture, and the internal generating power of capability (Stern et al., 1999).

The stable development of capability depends on the individual's degree of internal identification with the task. Therefore, whether an individual possesses the value associated with a task is a prerequisite for generating capability. Knowledge and experience are the necessary conditions for the generation and development of capabilities. However, if the individual does not possess values that are consistent with the task goal, even if the individual has perfect knowledge and rich experience, they will not be able to promote the generation of capabilities. Based on this, this article believes that values are the foundation of competence, and knowledge and experience run through the entire competence development process and are important influencing factors for competence development. In addition, feasibility judgments and effective response behaviors based on task recognition are important components of capability.

Capability is not innate. The generation of capability requires behavioral activities as the carrier, which follow the process of value generation, task identification, decision-making, feasibility prediction, and response. In general, there is an upward trend, and the lack of any link will affect the generation and advancement of capabilities. Only the balanced and orderly development of each link can continuously promote the capability to mature. The generation of capability depends on the continuous repetition of the behavior, so the mechanism of individual behavior needs to be considered when discussing the structure of capability. The theory of planned behavior (TPB) will facilitate our exploration of the dimensions of safety cognition capabilities of urban residents. TPB holds that individuals' behavioral decisions are influenced by their psychological characteristics and surroundings and other individuals' behaviors, which means that attitude toward behavior, subjective norm, and perceived behavioral control determine individuals' behavioral intentions. TPB concluded that behavior formation needs to go through three stages, namely, psychological foundation, behavioral intention, and behavior occurrence (Ajzen, 1991). The stage characteristics of behavior formation will contribute to constructing the conceptual structure of URSCC. It is worth noting that there is a significant difference between the capability of an individual to complete a task a single time and the capability to do so multiple times. Repetitive completion of a task will continuously improve the individual's capability. Generally speaking, the capability can gradually sublimate with the continuous development of the individual and eventually form a qualitative change, evolving into a high-quality capability. Therefore, an understanding of capability generation and evolution helps to further explain safety cognition capability.

Cognition can be regarded as a kind of psychological process, including many links, such as perception, thinking, information comparison, and implementation (Mesulam, 1998). After repeating these processes, cognition is transformed in an ascending spiral from the sensible to the rational, and finally to the practical (Stevenson, 2001; Gallese et al., 2004). Safety cognition capability is based on the concept of safe production and is put forward on the basis of general cognition capability. Safety cognition capability refers to people's attitudes, identification, judgment, and response to hazards in various purposeful activities. Previous studies on safety cognition capability are mainly concentrated in the fields of transportation, construction, and coal mines (Hu et al., 2009; Han et al., 2019; Zhang et al., 2020). All recognize the process characteristics of cognition; that is, that the main links of safety cognition include hazard perception, prediction, and response (Guo et al., 2019; Dumbaugh et al., 2020). Safety cognition capability is a special capability. Capability theory holds that the generation and development of any capability must repeat the dynamic process of value formation, information identification, result prediction, and specific response (Helfat and Peteraf, 2003). The safety cognition capability of urban residents also follows this development principle. The implementation of residents' safety behavior is the carrier of their safety cognition capability, which is a specific behavior. The generation of a fixed behavior pattern depends on the stable values driven by individuals, which is also supported by the theory of value-belief-norm (Stern et al., 1999). Safety values are an individual's sensible understanding of the safety climate and constitute the basic premise and core element of urban residents' safety cognition capabilities. In other words, safety values are the threshold level of urban residents' safety cognition capabilities, and the formation of safety values is the embryonic stage of safety cognition capability. After an individual has acquired mature safety values, the first step of safety cognition is the identification of various hazards, which we call the hazard source identification capability, referring to the capability of effectively identifying potential hazard sources after mastering safety knowledge and experience. Therefore, on the basis of the formation of safety values, if an individual has acquired the hazard source identification capability at the same time, this constitutes the perception level of the safety cognition capability of urban residents. The development from a safety value to hazard source identification capability is the formation stage of the safety cognition capability, and hazard identification source capability is a level of safety cognition capability. However, individuals who possess hazard source identification capability are not necessarily able to make safe behavioral choices (Neal and Griffin, 2006). Driven by safety values, individuals can make instantaneous and short-term hazard prediction through decision-making through their hazard source identification capability, which we call their hazard prediction capability. The safety cognition capability develops from the formation stage to the development stage, and the hazard prediction capability is the effective level of safety cognition capability. The progression theory of cognition points out that rational cognition is based on the accumulation of knowledge and the summary of one's experience, and the same is true for the generation and development of one's hazard source identification capability and hazard prediction capability, which constitute the rational stage of urban residents' safety cognition capability.

**Figure 1:**
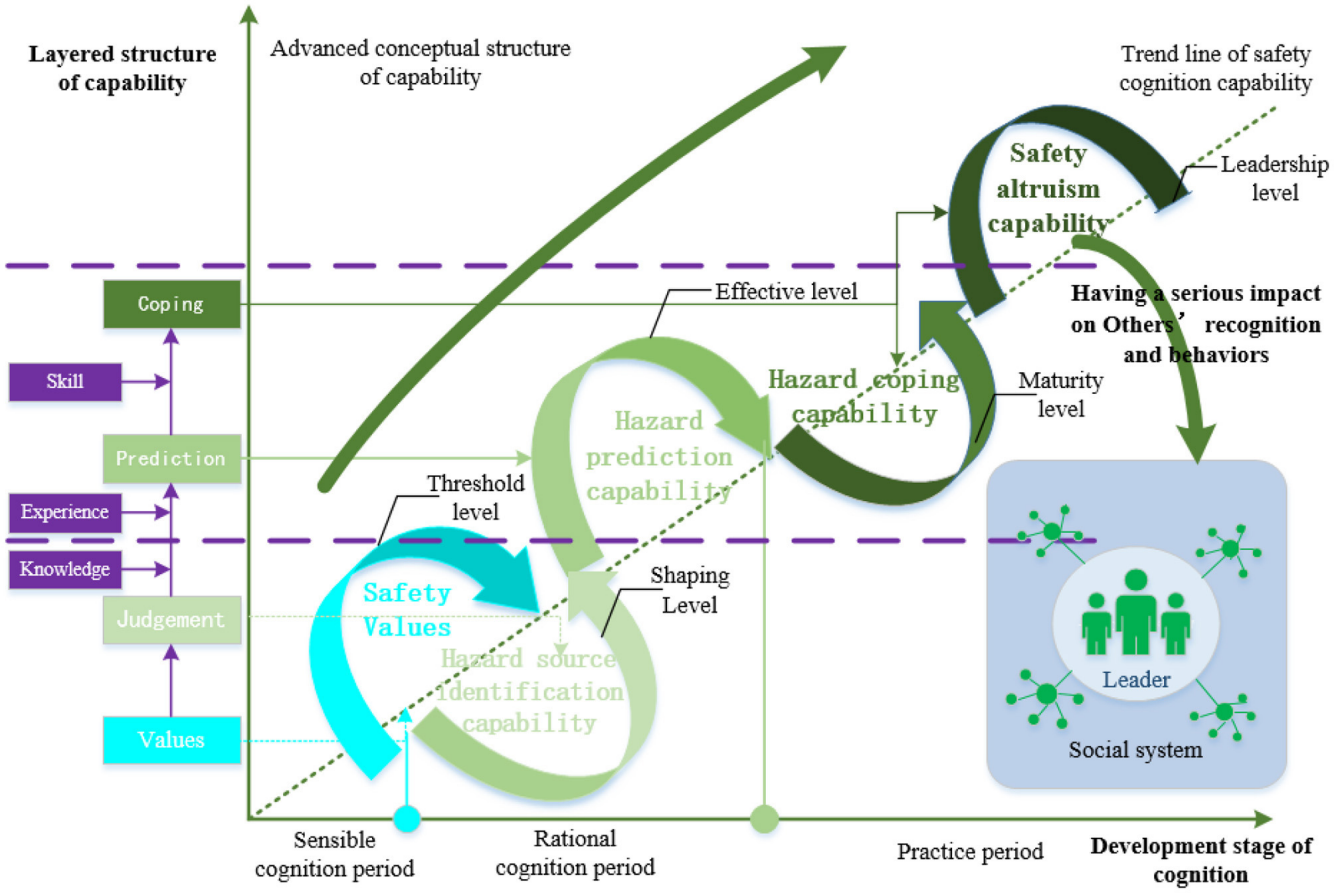
Conceptual structure of safety cognition capability in the whole process.

After predicting the hazards, the individual combines his own safety knowledge and experience to deal with it, that is, the individual uses their hazard coping capability. The evolution from the hazard prediction capability to the hazard coping capability indicates that the development of safety cognition capability has entered a mature period. In addition, safety cognition capability does not stop at individual safety behavior decision-making but spreads the safety values, knowledge, and experience to other people around the individual, who acts as a “missionary” and helps others as a coordinator in the face of public hazards (Jones-Lee, 1991). It can be seen that the linkage effect between the individual and the group, namely one's safety altruism capability, should also be paid attention to when exploring the structure of the safety cognition capability. Although the concept of the safety altruism capability has not been explicitly proposed by scholars, we found that emergency responses of individuals can be adjusted through social practices (Giddens, 1986). We found that safety altruism capability is an intangible but far-reaching safety belief that will continue to affect others and society as a whole. Safety altruism capability is a sublimation of individual capability, and it is the positive diffusion effect of influence between individuals, individuals and groups, and between groups. The performance of safety cognition capability should not stop at one's hazard coping capability but at safety altruism capability as the top level of safety cognition capability. Hazard response capability and safety altruism capability are effective behavioral responses to hazards and constitute the practical stages of the safety cognition capabilities of urban residents. Notable is that not all individuals follow the above capability development process. In real life, there are some individuals who have safety values but do not have hazard identification capabilities, but still show some hazard prediction capabilities, and even show some safety altruism capability, wherein the hazard forecasting and altruistic behavior is an accidental phenomenon with low power and extreme instability. In short, there exists a phenomenon of leapfrogging in some groups regarding urban residents' safety cognition capability. Based on the above analysis, we believe that the generation and development of safety cognition capability follows a process from self-capacity building to group-capacity diffusion, including safety values, hazard source identification capability, hazard prediction capability, hazard coping capability, and safety altruism capability, as shown in Figure 1.

## Measurement

Some representative studies on the dimensions and scales of safety cognition capabilities are shown in Table 1. Most of the existing studies have taken the safety cognition capability of specific groups in a single context as the object of study, and few researchers have focused on the URSCC in their daily life and work. In the field of food safety, a measurement framework based on three dimensions of self-reported behavior, psychosocial measures, and knowledge was pioneered (Altabbakh, 2013). Some researchers have argued that psychosocial measures do not fully explain individuals' internal perceptions and evaluations of safety and that self-reported behaviors only reflect some aspects of safety cognitions. Based on this, Byrd proposes to measure food safety cognition along three dimensions: attitude, knowledge, and self-efficacy (Byrd-Bredbenner et al., 2012). The scale developed by Chen is mainly used to measure the safety cognition of construction workers, including human error, safety performance, accident causes, risk perception, management actions, safety management and control, as well as accident statistics, totaling 29 items (Chen et al., 2011). In recent years, several researchers have explored the structure of individuals' safety cognition from the perspective of general abilities. For example, Guo argues that individuals' attention, multiple reaction ability, learning ability, short-term memory, and performance stability constitute their safety cognition capabilities (Guo et al., 2019). Han combines personal and external factors and divides safety cognition into three dimensions: implicit social cognition, explicit social cognition, and outer-layer artifacts (Han et al., 2019). Some researchers have also taken the influence of experience on safety cognition into account by including whether individuals have received safety training as a dimension of safety perceptions. For example, Altabbakh developed a scale to measure safety training, safety knowledge, safety attitude, and safety consciousness (Altabbakh, 2013). The scale developed by Li and Li includes factors, such as on-site hazard identification, worker risk behavior identification, occupational safety, and regulatory understanding of site hazard identification and regulations (Li and Li, 2017).

**Table 1:** Safety cognition capability dimension.

**References**	**Dimension**	**Research object**
Abbot et al. (2009)	Self-reported behaviors, psychosocial measures, knowledge	Adolescent
Chen et al. (2011)	Human error, safety performance, accident causes, risk and perception, management actions, safety management and control, accident statistics	Employee
Han et al. (2019)	Implicit social cognition, explicit social cognition, outlet layer artifacts	Employee
Altabbakh (2013)	Safety training, safety knowledge, safety attitude, safety consciousness	College student
Byrd-Bredbenner et al. (2012)	Knowledge, attitudes, self-efficacy.	Middle schoolers
Guo et al. (2019)	Attention, multiple reaction ability, learning ability, short-term memory, performance stability.	Employee
Li and Li (2017)	On-site hazard identification, worker risk behavior identification, occupational safety, regulatory understanding	Employee

In terms of an applicable situation, the existing scales mainly focus on the workplace and, as a result, their application areas and situations are restricted. Aside from that, the study focus of other scales has been varied but scattered, with a low degree of recognition and a limited application area and situation. In addition, the URSCC has been enriched through the development of society, and the existing literature is deficient in terms of comprehensive indicators that respond to psychological and individual-group connections. Due to the lack of measurement tools for precision and operability, these scales cannot be directly applied to describing the safety cognition capability of urban residents. Despite these disadvantages, such studies are valuable resources that have led to the development of our scale. We referred to the dimensional settings and statements from the previous scales and modified our self-developed questions with the relevant measurement statements from these scales. For example, for the items of the URSCC scale that relate to the public health domain we refer to this statement: “I eat: raw oysters, clams or mussels, rare hamburgers, raw homemade cookie dough or cake batter, sushi.” We replaced the foods mentioned in the statement with foods that are more preferred by Chinese urban residents to ensure the localization of the scale.

Furthermore, grounded theory stresses the use of original data and bridges the gap of theory and reality through methods including literature reviews, interviews, and coding, which can successfully solve flaws in past research in this field. As a result, based on substantial literature research, the URPS scale was developed using a combination of qualitative and quantitative methodologies. We developed the initial scale using grounded theory, and we statistically analyzed the structure of the URPS scale using data acquired from questionnaires.

### Initial Scale Construction

To extract the initial question items of the URSCC scale, we conceptualized the specific performance characteristics of the URSCC. We also obtained the initial question items by (1) conducting interviews with selected urban residents and compiling and editing the interviews, and (2) Literature analysis and in-depth analysis of studies on safety cognition and capability evolution to provide a theoretical basis for the scale development.

In addition, these interviews were conducted on the basis of a simple outline that did not include predetermined paradigms and assumptions but which was used as an aid to guide the interviewees' recall and description of the questions, as detailed in Table 2. The questions were explained to the interviewees before the interview and could be adjusted during the interview according to the actual situation to elaborate on the topics of the interviewees' responses.

**Table 2:** Outline of interview on the safety cognition capability of urban residents.

**Theme**	**Main content**
Basic information	Gender, age, education level, address, income level, work level, nature of organization
The current situation of urban residents' sense of safety	a. Do you think it is important to be safe? b. How do you feel about the city you live in?
The structure of URSCC	a. What capabilities do you think are necessary to ensure the safety of yourself and others around you? b. Have you encountered certain risks? And tell us how you responded to them? c. Are there any groups around you that are particularly safety conscious? Describe what qualities they all have in common?

Grounded theory requires that the research subjects are in different age groups, have different education levels, different occupations, and income levels. Therefore, we selected 30 respondents through online publicity. The process of selecting interviewees was carried out according to the theoretical sampling procedure of the grounded theory research method. The method of purposive sampling was used to invite urban residents from different regions as respondents through social software. Considering different cultural backgrounds and regional differences, different groups in eastern, central, and western cities were selected as research participants, including Hebei Province and Jiangsu Province in the eastern region, Anhui Province and Hunan Province in the central region, and Sichuan Province and Xinjiang Province in the western region, giving full consideration to the representativeness of the sample. The basic descriptive statistics of the respondents are as follows: 53% are male and 47% female, 40% are 21–30 years old, 47% are 31–40 years old, and 13% are over 40 years old; 60% of the respondents have a bachelor's degree. In addition, respondents were from cities of different sizes and had different income levels. Details of the interviewees are shown in Appendix B. We transformed a representative sample of recordings into text, totaling 42,000 words. In addition, we conducted a theoretical saturation test, which means that when the information obtained from the interview begins to repeat itself and no new important information emerges, the results of the interview have reached theoretical saturation and no further interviews are needed (Glaser and Strauss, 2017). However, the five randomly selected respondents did not provide any new information, which shows that the interview content is theoretically saturated. We invited six researchers to organize the interview texts and collect words and phrases related to safety cognition capabilities. The original statements were then further integrated and simplified by combining them with the literature review.

After initial sorting and categorization, 239 original statements about “safety cognition capability” were collected. Six researchers coded and labeled these expressions and then iteratively discussed them, removing 63 of them that were ambiguous. In view of the diversity of the remaining 176 expressions, we simplified and generalized them based on the analysis of the literature to form specific conceptual indicators. The specific results are summarized in Table 3.

**Table 3:** Classification of semantically similar items.

**Original statements**	**Conceptualization**	**Frequency**
A safe atmosphere is the basic guarantee for my daily life and work; I am willing to preach some safety knowledge; I think a safe atmosphere in the city needs everyone's joint efforts.	Safety values	35
I think the city I live in has more bad weather, serious environmental pollution, frequent man-made accidents, unsafe food, widespread occupational diseases, frequent infectious diseases, unsafe network, and no guarantee of personal safety.	Natural disasters, accidents and disasters, public health events, social security events	30
I think knowing the common hazards can avoid some risks. For some uncertain things, I will understand to ensure their own safety.	Identification of hazard source	21
When I receive a call from an unfamiliar caller and money is involved, I will be vigilant; When buying bagged food, I will pay attention to the date of manufacture and production license.	Hazard prediction	21
When I was followed by a stranger, I quickly moved to a convenience store while calling my family; When I encountered an agitated passenger grabbing the steering wheel on a bus, I stopped it in time; I work in the restaurant industry and can often identify foods that have hygiene problems; When a fire broke out at work, I knew how to use the fire extinguisher and put out the fire in time; Once when a typhoon passed through, I ran to the open outdoor area and was not hit by the collapsed house.	Hazard coping	14
I am surrounded by groups of people who specialize in safety management, who have a high level of safety awareness, are able to anticipate hazards, and are able to correct unsafe behavior in the groups around them; My beloved is a firefighter and often stresses safety awareness to me, and he always handles emergencies appropriately when he encounters them.	Influence and command	11

An individual's level of safety cognition is influenced by various factors, such as their knowledge base, occupation, and social background. The strength of risk perception varies among residents of different social backgrounds (Zhang et al., 2021). It was found that gender, age, ethnicity, education, wealth, job hierarchy, nature of the unit, intelligence, and prestige all have an impact on individuals' safety cognition capability. Based on the collated entries and literature review, we concluded that “gender,” “age,” “education,” “monthly income,” and “job level” of urban residents are related to the level of their safety cognition capability, and five questions were formed.

Attitudes toward ideals, customs, and social norms are collectively referred to as values (Aiken, 2010). Values determine individual attitudes, that is, the “stable emergence” of capabilities depends on the individual's internal identification with the task and is essentially determined by a high degree of alignment between values and task goals. Safety cognition capability is a special kind of capability that also follows this rule. We believe that safety cognition capabilities originate from stable safety values, that is, the active maintenance of one's own safety and that of others. Similar expressions were found in the collected entries, such as “safety is a basic guarantee,” being “willing to spread safety knowledge,” “maintain public safety,” and “stop dangerous behavior.” This led to the compilation of three scale questions.

The collation revealed that some of the terms were related to urban residents' levels of knowledge about the sources of hazards. The dangers perceived by residents are multifaceted (Yibao Wang et al., 2018), such as “more severe weather (natural disasters),” “frequent man-made accidents (accidents and disasters),” “unsafe food (public health events),” and “life safety is sometimes not guaranteed (social safety events),” which are all sources of hazards that urban residents are exposed to on a daily basis. In addition, we note that many of the collected phrases emphasize the positive effects of having the capability to identify hazards, such as “knowing common hazards can avoid some risks,” and that hazard identification is an important part of safety cognition, resulting in 10 scale items.

The study found that the capability to predict risks is an important part of effective safety cognition and that shortening the psychological distance from risk can motivate individuals for this kind of cognition. Combining the frequency of the words and the existing research, the questions of “knowing the level of disaster warning,” “being able to recognize the main symptoms of infectious diseases,” “being able to recognize crowded people where a trampling accident may occur” were categorized as “hazard prediction capability,” and 10 questions were developed.

It has been noted that practice is part of cognition and can correct for biases. Successful risk avoidance experiences can deepen an individual's attitude and understanding of safety, that is, hazard coping is an integral part of safety cognition. According to the collated entries and existing research, the capability to “use fire extinguishers correctly,” “getting away from strangers quickly at any time,” “knowing how to respond when typhoons pass,” and the capability to “distinguish unsanitary food” are attributed to the urban residents' “hazard coping capability” and formed 10 measurement items.

In addition, “I can command and coordinate people around me to deal with danger” appeared three times. The use of this capability should not stop at the individual but has a diffusion effect when it occurs in a broad social group. “I can influence the attitude of the group around me toward safety” and “I can command and coordinate others to respond to hazards” reflect the externalization of individual safety cognition in the group, resulting in the development of three scale items.

The specific steps of the grounded theory analysis include open coding, axial coding, and selective coding (Corbin and Strauss, 1990). During the open coding phase, the researchers debated the statements multiple times before deciding to reclassify them based on semantic similarity and eliminate ambiguous items, leaving 176 statements. Considering the complexity of the remaining 176 statements, at the axial coding step, the researchers integrated and simplified them to create conceptual indicators based on the literature review. Selective coding is a continuation of axial coding at a higher level of abstraction to find out the core category. We followed this criterion to summarize the proposed conceptual indicators, eventually forming a URSCC scale, consisting of 41 items. The purpose of this study is to enhance the theoretical logic and content validity of the structural system of the URSCC through a qualitative research method. In the following section, we will use quantitative analysis to further examine and revise the structural system through data.

## Quantitative Method

### Preliminary Survey and Extraction of the URSCC Scale

After the initial completion of the URSCC scale, the validity and reliability of the initial scale needed to be analyzed before the formal scale was formed by revising some of the questions. First, through random sampling, researchers promoted and disseminated the web link to the online questionnaire on social media platforms and expanded the number and scope of respondents by continuously forwarding the link. Secondly, in order to make the distribution of the surveyed population reasonable in terms of demographic characteristics, a stratified random sampling method was used to distribute some questionnaires with the help of a professional questionnaire survey website in China. Finally, we compared the selected demographic data with nationally representative demographic data. The demographic data of the survey sample matched well with the national demographic data. At the same time, to ensure the active participation of residents, we provided cash rewards for completing the questionnaire. The preliminary survey was started on 4 February 2020, and a total of 298 questionnaires were collected, of which 53 samples were excluded due to the selection of the same answer for multiple consecutive questions, so that 245 valid questionnaires were obtained, with a valid questionnaire recovery rate of 82.2%. The number of preliminary survey subjects should be three to five times the maximum number of subscale items in the entire scale, and the larger the sample, the better the scale test (DeVellis and Thorpe, 2021). Therefore, the sample size of the preliminary survey should be greater than 30, and the sample size was in line with the standard for scientific research.

First, we conducted reliability tests on the initial scales. Cronbach's alpha coefficient was used to determine the overall reliability of the scale. The results showed that the Cronbach's α value of the URSCC scale was 0.793, indicating that the overall reliability of the scale was acceptable. Item analysis was used to determine the reliability of each item in four ways: (1) Descriptive statistical analysis: Descriptive statistics for each item were used to assess the basic quality of the item, and there were no low discrimination items with standard deviations of less than 0.75. (2) Extreme group test: Among the 298 residents surveyed, we selected 27% of the highest total scores and 27% of the lowest total scores and conducted independent sample *t*-tests for the extreme groups. The *t*-test values all reached a significance level of 0.05, indicating that each item was effective in identifying high and low scores. (3) Correlation test: Of the 41 questions on the scale, all were significantly correlated with the total score on the scale. (4) Cronbach's α value test: The data showed that the overall reliability of the scale decreased when any of the entries were removed. Thus, 41 items remained in the URSCC scale after item analysis. We conducted a component analysis of these 41 items. During testing, we removed any items with factor loading values of less than 0.5 or with cross-loading values greater than 0.4. After a multi-factor analysis, items 7, 19, and 29 were deleted, and a better discriminant factor structure was obtained. Finally, based on feedback from some respondents and discussions with experts, the linguistic expression of the scale items was improved, thus, further improving the accuracy and clarity of the scale expression and the content validity of the scale summary. We also improved the quality of the initial scale by conducting a pre-study assessment and a formal survey. The final URSCC scale consists of 38 items. The scale was used for the formal research.

### Formal Survey and Structural Analysis of the URSCC Scale

The formal investigation was launched in March 2020, and a total of 793 samples with 735 valid survey responses were obtained. For factor analysis, the ratio of the number of items per question to the sample size ranged from approximately 1:5 to 1:10, which was not as important if the total number of subjects was 300 or more (Tinsley and Tinsley, 1987). The structural distribution of the sample is shown in Table 4. SPSS20.0 and AMOS16.0 were used to analyze the questionnaire data. The specific analysis is shown in Table 4.

**Table 4:** Sample distribution.

**Social demographic**	**variables**	**Frequency**	**Percentage**	**Social demographic**	**variables**	**Frequency**	**Percentage**
Gender	Male	404	54.97	Age	20 and below	20	2.72
	Female	331	45.03		21–25	181	24.63
Education	Junior high school and below	17	2.32		26–30	232	31.56
	High School/technical school	66	9.01		31–40	155	21.09
	Junior college	83	11.34		41–50	128	17.41
	Undergraduate	419	57.02		51 and above	19	2.59
	Master's degree	141	19.19	Monthly income(RMB)	<2,000	146	19.82
	Ph.D.	8	1.12		2,000–4,000	185	25.11
Job level	Entry level employee	338	45.95		4,000–6,000	120	16.3
	Grassroots management	158	21.45		6,000–8,000	145	19.86
	Middle management	58	7.93		8,000–10,000	105	14.22
	Senior management	26	3.52		10,000–30,000	22	3.05
	Other	155	21.15		30,000–100,000	12	1.64

#### Exploratory Factor Analysis

An exploratory factor analysis was performed on half of the sample (*N* = 368) using SPSS 20.0. The KMO value of the scale was 0.909 > 0.8, and the significance level passed Bartlett's test (*p* < 0.001), indicating that the scale could be subjected to factor analysis. The factor loading matrix was then obtained through principal component analysis and an orthogonal rotation method. As shown in Table 5, we selected five eigenvalues greater than 1 based on the Kaiser criterion, with a cumulative variance explained of 60.169%. The definition of each factor is shown in Table 6.

**Table 5:** Exploratory factor analysis results.

**Item**	**Communality**	**Factor**			**Item**	**Communality**	**Factor**	
		**S4**	**S3**	**S5**			**S2**	**S1**
URSCC-3	0.753	0.863			URSCC-22	0.579	0.738	
URSCC-2	0.751	0.860			URSCC-18	0.583	0.735	
URSCC-1	0.790	0.843			URSCC-21	0.532	0.718	
URSCC-6	0.747		0.780		URSCC-14	0.573	0.695	
URSCC-13	0.658		0.771		URSCC-16	0.607	0.685	
URSCC-5	0.638		0.770		URSCC-17	0.418	0.679	
URSCC-9	0.542		0.732		URSCC-23	0.578	0.656	
URSCC-11	0.593		0.721		URSCC-15	0.620	0.655	
URSCC-8	0.511		0.714		URSCC-20	0.492	0.614	
URSCC-10	0.582		0.704		URSCC-33	0.603		0.816
URSCC-12	0.494		0.664		URSCC-26	0.563		0.747
URSCC-4	0.612		0.615		URSCC-24	0.628		0.726
URSCC-36	0.703			0.803	URSCC-30	0.507		0.710
URSCC-34	0.684			0.742	URSCC-32	0.501		0.698
URSCC-35	0.739			0.735	URSCC-25	0.580		0.690
					URSCC-28	0.403		0.677
					URSCC-27	0.556		0.629
					URSCC-31	0.735		0.552

**Table 6:** Definition of each factor.

**Factor**	**Definition**
Safety value	Urban residents' attitude, view and internal recognition of safety
Hazard source identification capability	Urban residents' understanding of hazard sources in various fields
Hazard prediction capability	Urban residents' capability to accurately predict danger scenes
Hazard coping capability	Urban residents' capability to continuously and stably effectively deal with various dangerous situations in their daily life and work practice.
Safety altruism capability	Urban residents' capability to influence people around them to improve their safety cognition capability in words or actions

#### Confirmatory Factor Analysis

Using the other half of the data (*N* = 367), the conceptual model obtained by exploratory factor analysis was tested for its fit to the actual observed data. To better verify the accuracy of the model, five competing models are proposed below for comparison with the results of the model obtained from the exploratory factor analysis.

- M1: One-factor model, assuming that the common latent variable embraced by the 33 questionnaire items is the URSSC.
- M2: Two-factor model, assuming that 12 items measuring safety values and hazard source identification ability have common latent variables, and 21 items of hazard prediction capability, hazard coping capability, and safety altruism ability have common latent variables.
- M3: Three-factor model, assuming that there are common latent variables for 12 items measuring safety values and hazard source identification capability, 18 items measuring hazard prediction capability and hazard coping capability, and 3 items measuring safety altruism capability.
- M4: Four-factor model, assuming that 12 items measuring safety values and hazard source identification capability have common latent variables, 9 items measuring hazard prediction capability have common latent variables, 9 items measuring hazard coping capability have common latent variables, and 3 items measuring safety altruism capability have common latent variables.
- M5: The five-factor model, based on the results of exploratory factor analysis, assumes five factors for safety values, hazard source identification capability, hazard prediction capability, hazard coping capability, and safety altruism capability.

For each of the above models, the validated factor analysis was conducted with each factor as the latent variable and its corresponding question item as the observed variable. The model fitting results are shown in Table 7. The fit results of M1, M2, M3, and M4 are not satisfactory, and the GFI and AGFI of all four models are less than 0.7, while NFI, CFI, TLI, and IFI are less than 0.9, and RMSEA is greater than.07. The χ^2^/df of M5 model is 2.828, which is the smallest of the remaining five models, and CFI, TLI, and IFI are all greater than 0.9, so that the first-order model M5 is optimal. However, there are still some indicators that have not reached an excellent level. Once the model parameters were corrected, the correction index was greater than 20 variance coefficients collated, (see Table 8).

**Table 7:** Major fitting degree indices of URSCC.

**Model**	**χ^2^**	**df**	**χ^2^/df**	**GFI**	**AGFI**	**NFI**	**CFI**	**TLI**	**IFI**	**RMSEA**
M1: Single-factor model	6732.452	805	8.363	0.514	0.449	0.503	0.521	0.489	0.522	0.131
M2: Double-factor model	4933.298	804	6.136	0.677	0.633	0.636	0.659	0.636	0.660	0.111
M3: Triple-factor model	4714.427	803	5.871	0.682	0.639	0.652	0.676	0.654	0.677	0.088
M4: Four-factor model	3128.957	799	3.916	0.793	0.798	0.788	0.856	0.834	0.799	0.066
M5: Five-factor model	2254	797	2.828	0.856	0.889	0.851	0.903	0.902	0.909	0.059

**Table 8:** Overall fitting degree indices of each modification.

**Title**		**Initial model fitting**	**Release 24-e30**	**Release e14-e22**	**Release e11-e12**	**Assessment**
Absolute fitting index	χ^2^	2254.216,d f = 797 *P* = 0.000	2243.274, df = 795 *P* = 0.000	2237.125, df = 793 *P* = 0.000	2231.437, df = 792 *P* = 0.000	Great
	GFI	0.856	0.881	0.897	0.909	Great
	RMR	0.066	0.064	0.064	0.061	Good
	RMSEA	0.059	0.057	0.051	0.045	Great
Relative fitting index	AGFI	0.889	0.894	0.899	0.901	Great
	NFI	0.851	0.862	0.871	0.888	Good
	TLI	0.902	0.917	0.922	0.931	Great
	CFI	0.903	0.907	0.911	0.919	Great

**Figure 2:**
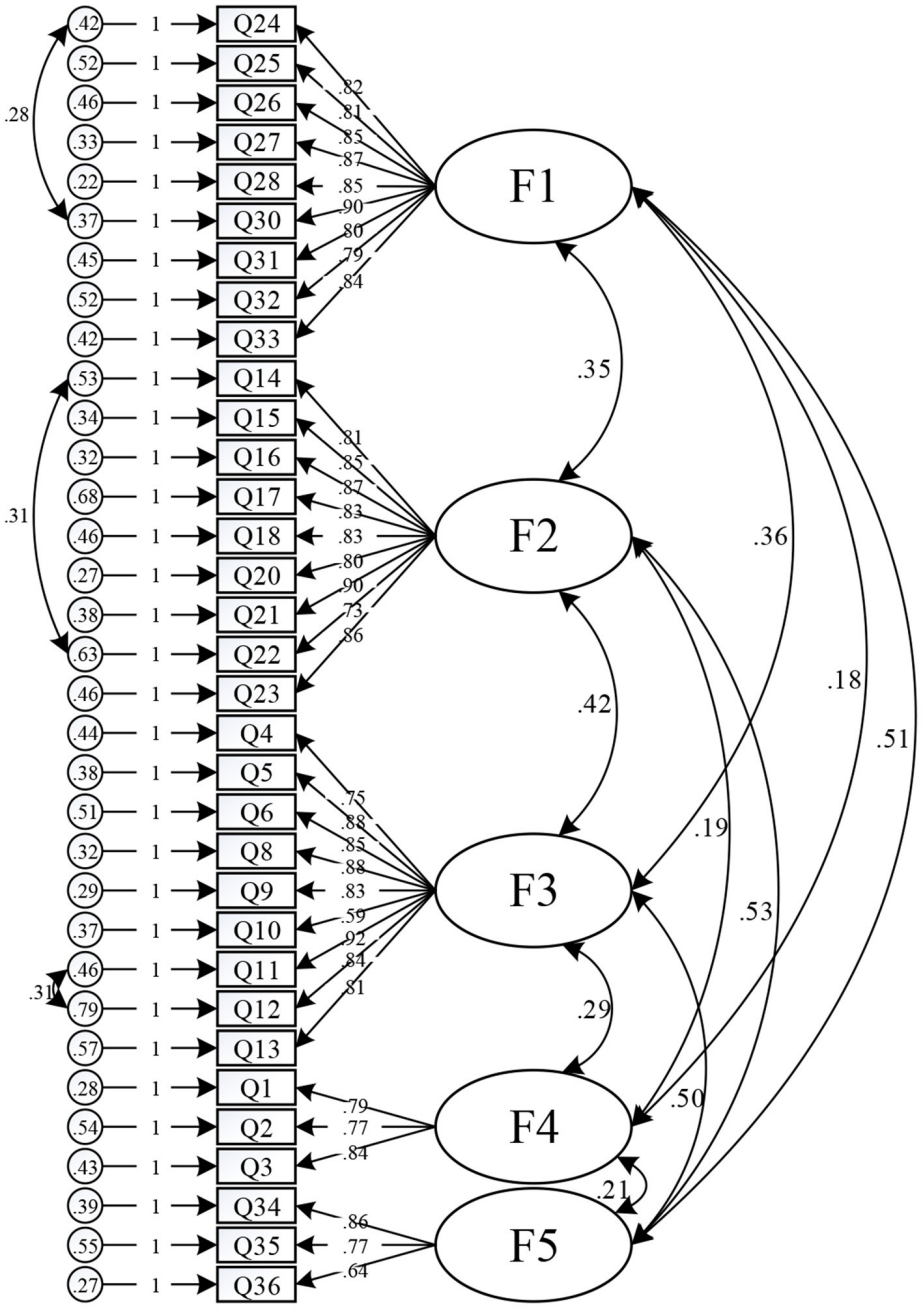
Estimations of the standardized path coefficient of the final confirmatory factor model.

After three model corrections, the GFI, AGFI, TLI, and CFI values were all greater than 0.9, the RMSEA value was below 0.05, and the χ^2^/df value was 2.817. All indicators reached a good range, showing that the model of URSCC has an ideal fit. The standardized path diagram is shown in Figure 2.

#### Reliability and Validity

The assessment of the scale reliability mainly includes two levels: the overall reliability of the scale and the reliability of the latent variables, in which the Cronbach's α value (>0.7) was used to test the overall reliability of the scale, and the Cronbach's α value and CR (>0.6) were used to test the reliability of the latent variables. After analyzing the data, it was found that the overall Cronbach's α value of the URSCC scale was 0.928, and that the scale was, thus, reliable as a whole. The Cronbach's α values of the latent variables ranged from 0.799 to 0.901, and the CR values were all above 0.7, both of which were above the acceptable standard, indicating that the scale passed the reliability test.

The assessment of scale validity mainly includes two aspects: content validity and structural validity, in which content validity is mostly measured with qualitative methods, and the validation of structural validity mainly examines the convergent validity and discriminant validity of the scale. In this paper, the initial questionnaire was developed in strict accordance with the scale development procedure, based on a large number of prior studies, and five domain experts were invited to discuss the questionnaire design repeatedly. In total, 298 pre-surveys were conducted, so the content validity of this scale is reliable. In addition, the standardized loadings of the 33 items of the scale on the corresponding latent variables were all greater than 0.5 and reached the significance level, and the corresponding AVE values ranged from 0.581 to 0.701, which satisfied AVE > 0.5, indicating that the convergent validity of the scale was good. In addition, the square roots of the AVEs of the latent variables were all greater than the correlation coefficients between the latent variables, indicating that the potential structural differentiation of the variables was good. The scale passed the validity test. The specific analysis is shown in Table 9.

**Table 9:** Reliability and validity test of each factor.

	**F1**	**F2**	**F3**	**F4**	**F5**
F1	0.889^*^				
F2	0.494	0.832^*^			
F3	0.619	0.346	0.822^*^		
F4	0.488	0.447	0.336	0.801^*^	
F5	0.557	0.352	0.452	0.395	0.762^*^
Cronbach'sα	0.897	0.894	0.901	0.845	0.799
CR	0.955	0.953	0.949	0.824	0.804
AVE	0.701	0.693	0.675	0.641	0.581

* *indicates the square root of the AVE value*.

## Discussion and Conclusion

### Discussion

The URSCC scale measures the safety cognition capability of urban residents regarding the dimensions of safety values, hazard source identification capability, hazard prediction capability, hazard coping capability, and safety altruism capability with objectivity, which truly and clearly reflect the level of urban residents' safety cognition.

The capability theory argues that the emergence and development of any capability follows a dynamic process of value formation, information recognition, outcome prediction, and concrete response, which is repeated over and over again (Wei et al., 2016). Cognition consists of four main processes: information reception, initial analysis, strategy selection, and concrete implementation (Wickens, 1984). In a study of construction workers' cognitions of unsafe behaviors, some scholars have proposed a model of safety cognition that includes four components: hazard identification, reasoning and analysis, decision generation, and implementation response (Goh and Sa'Adon, 2015). This study proposes a safety cognition capability model for urban residents based on the capability theory and the safety cognitive process model.

In addition, this research innovatively proposes two dimensions of safety values and safety altruism capability based on a large number of interviews.

Altabbakh (2013) argues that safety attitudes and awareness are important components of safety cognition capability. However, awareness and attitudes are only the external manifestations of an individual's internal identity, while values are the ultimate origin of behaviors and are the intrinsic motivation for the generation of individual capability. The generation of fixed behavioral patterns depends on stable values within the individual, which is also supported by the “value-idea-norm” theory (Stern et al., 1999). Few researchers have considered the diffusion effect of individual safety cognition capability in groups when developing safety cognition capability scales because the proposed safety altruism capability dimension can also be considered as an innovative contribution of this paper.

In terms of scale applicability, most of the existing scales are applicable to a single context, such as health care settings (Feng et al., 2021), construction sites (Trillo-Cabello et al., 2020), driving (Farrand and Mckenna, 2001), or natural hazards (Crescimbene et al., 2015; Eryilmaz Türkkan and Hirca, 2021). There is no scale that specifically measures the URSCC, and the URSCC scale can be mainly applied to situations that are relevant to the daily lives of urban residents, specifically natural disasters, accidents, public health events, and social security events. In addition, although the survey was conducted in China, the scale is applicable not only to developing countries that have achieved rapid economic growth at the expense of the environment, such as China and India but also to developed countries with strict environmental requirements, such as countries of the European Union and the United States.

### Conclusion

First, we conducted in-depth interviews with 30 respondents and developed a URSCC scale consisting of 41 items through qualitative analysis on the basis of existing related studies. Then, we obtained 245 samples through a pre-study, and based on this, we used item analysis and principal component analysis to purify and validate the structure of the scale to finally form a formal research scale of URSCC, consisting of 38 items.

A total of 735 questionnaires was obtained from the formal survey, and we used half of the sample for a principal component analysis to obtain the following six factors: “safety values,” “hazard source identification capability,” “hazard prediction capability,” “ability to respond to hazards,” and “safety altruism capability.” The KMO of the scale was 0.90, which is greater than 0.7, and the significance was 0.000. The cumulative variance of the six factors was 60.169%. We performed a validated factor analysis of the scale using the other half of the data, and the results showed that the M5 model was superior to the other four models. In addition, we corrected the model parameters because some of the indicators did not meet the requirements. The corrected model had an RMSEA value of 0.059, a χ^2^/df value of 2.828, and GFI, AGIF, TLI, CFI, NFI values of 0.856, 0.889, 0.902, 0.903, and 0.851, respectively. The indicators reached the desired range, indicating that the URSCC model has a good fit.

Finally, the reliability of the scale was examined. The Cronbach's α value of the overall reliability of the URSCC scale was 0.928, which is higher than 0.7, and the Cronbach's α values of each latent variable were 0.897, 0.894, 0.901, 0.845, and 0.799, respectively. The CR values were 0.955, 0.953, 0.949, 0.824, and 0.804, respectively. The CRs were 0.955, 0.953, 0.949, 0.824, and 0.804, all of which were within a reasonable range, and the scale had good reliability. In addition, the development of the scale was carried out in strict accordance with the procedures, and the process was rigorous and scientific, which ensured the reliability of the content validity. The standardized loadings of the 33 items of the scale on the corresponding latent variables were all greater than 0.5, and the corresponding AVE values were 0.701, 0.693, 0.675, 0.641, and 0.581, all of which were greater than 0.5. The convergent validity of the scale was also good, and the square roots of the AVEs of the latent variables were greater than the correlation coefficients between the latent variables. The potential structural differentiation of the variables was good, and thus, the scale passed the validity test.

### Limitations and Future Studies

The main limitations of this study are as follows: (1) There are local limitations in the sample. During the sampling process, we took the unevenness of urban development levels in China into account, and although the sample was selected to reflect most demographic variables, there were still some areas that could not be covered, and there was no difference in the scales used in cities with different development levels. (2) Since the study focused on urban residents, a large number of rural residents who completed the questionnaire had to be removed, resulting in a lack of comparative analysis of urban and rural residents. (3) The main contribution of this study is the development of the URSCC scale, which has not been empirically tested. Therefore, it is necessary to further validate, revise, and improve the scale. The validity of the scale has only been verified in China. We expect to use this scale to measure and compare the safety cognition capabilities of urban residents in different countries and cities in the future, validating the applicability of the URSCC scale in different countries and regions. Next, we will conduct a large sample survey using the URSCC scale. Then, based on the sample data, we plan to analyze the differences in dimensions and variables across regions to determine whether there are significant differences in the effects of economic development, social development, and technological development on the five main factors in different regions. Meanwhile, studies were conducted in the areas of urban mobility rate, regional integration, and urban crime rates, using the perceived safety capacity of urban residents as a mediating variable.

## Data Availability Statement

The raw data supporting the conclusions of this article will be made available by the authors, without undue reservation.

## Ethics Statement

The study protocol was reviewed and approved by the Ethics Committee at the Department of Administration, China University of Mining and Technology. The patients/participants provided their written informed consent to participate in this study.

## Author Contributions

YX obtained the data and wrote the article. CZ conceived the study and performed the field research. HQ contributed to the research design and the data collection. RZ and YZ provided advice for revisions. All authors contributed to the article and approved the submitted version.

## Funding

This study was funded by the National Natural Science Funding of China (No. 71303233), the Social SciencePlanning Project of Jiangsu Province (No. 18ZZB001), and the Fundamental Research Funds for the Central Universities (No. 3142018050).

## Conflict of Interest

The authors declare that the research was conducted in the absence of any commercial or financial relationships that could be construed as a potential conflict of interest.

## Publisher's Note

All claims expressed in this article are solely those of the authors and do not necessarily represent those of their affiliated organizations, or those of the publisher, the editors and the reviewers. Any product that may be evaluated in this article, or claim that may be made by its manufacturer, is not guaranteed or endorsed by the publisher.
