# Supplementary material for: How to Measure the Safety Cognition Capability of Urban Residents? An Assessment Framework Based on Cognitive Progression Theory
Source: Front Psychol. 2022 Apr 12;13:707172. doi: 10.3389/fpsyg.2022.707172 (PMC9039215; doi:10.3389/fpsyg.2022.707172)
Supplement: Supplementary file 1 [file Table_1.pdf]

Appendix

Appendix A The concept of five dimensions of URSCC

Term	Concept definition
Safety values	Safety values refer to urban residents' attitudes, views, and internal recognition of safety, which is the intrinsic driving force and value foundation of a series of behaviors of urban residents to deal with hazards.
Hazard source identification capability	Hazard source identification capability refers to urban residents' understanding of hazard sources in various fields, including both actual and potential hazard sources.
Hazard prediction capability	Hazard prediction capability refers to the urban residents' capability to flexibly use their own knowledge and experience to accurately predict existing or potential danger scenes when they are dealing with various hazard sources in specific situations.
Hazard coping capability	Hazard coping capability refers to residents' capability to continuously and effectively deal with various dangerous situations in their daily life and work. This coping capability is based on residents' long-term identification of risk sources and continuous avoidance of them in practical activities, which can be achieved through repetition.
Safety altruism capability	Safety altruism capability means urban residents' capability to influence people around them to improve their safety cognition capability through words or actions on the basis of reaching a mature level. This kind of influence capability is invisible, which is a social extension of the safety cognition capability of urban residents. It subtly influences the safety cognition capability of surrounding individuals and even society as a whole.

Appendix B Basic information of the participants

Number	Interviewee	Age	Education	Region	Job
R01	Mr. Shao	27	Master’s Degree	Hebei	Civil Servant
R02	Ms. Zhou	58	Junior high school and below	Hebei	Freelancer
R03	Mr. Zhen	33	Bachelor’s Degree	Hebei	Restaurant Manager
R04	Ms. Li	24	Bachelor’s Degree	Hebei	Company employee
R05	Ms. Qian	25	Junior College	Hebei	Company employee
R06	Mr. Chen	28	Senior High School	Jiangsu	Freelancer
R07	Mr. Wang	61	Junior high school and below	Jiangsu	Janitorial worker
R08	Mr. Geng	31	Master’s Degree	Jiangsu	University Faculty
R09	Mr. Zhao	21	Bachelor’s Degree	Jiangsu	College Student
R10	Ms. Wei	30	Bachelor’s Degree	Jiangsu	Civil Servant
R11	Ms. Zhang	37	Senior High School	Anhui	Company employee
R12	Ms. Zhang	30	Master’s Degree	Anhui	Counselor
R13	Mr. Cui	56	Senior High School	Anhui	Company employee
R14	Ms. Zhang	44	Bachelor’s Degree	Anhui	Secondary School Teacher
R15	Ms. Li	40	Bachelor’s Degree	Anhui	Civil Servant
R16	Mr. Zhao	37	Senior High School	Hunan	Company employee
R17	Ms. Li	35	Master’s Degree	Hunan	Doctor
R18	Ms. Zhao	28	Junior high school and below	Hunan	Company employee
R19	Ms. Han	32	Bachelor’s Degree	Hunan	Civil Servant
R20	Mr. Zhu	38	Bachelor’s Degree	Hunan	Company Manager
R21	Mr. Zhang	36	Senior High School	Sichuan	Company employee
R22	Mr. Li	33	Master’s Degree	Sichuan	University Faculty
R23	Ms. Hou	21	Bachelor’s Degree	Sichuan	College Student
R24	Mr. Wu	39	Junior College	Sichuan	Driver
R25	Ms. Ding	24	Master’s Degree	Sichuan	Graduate Student
R26	Mr. Cao	29	Junior high school and below	Xinjiang	Janitorial worker
R27	Mr. Xu	26	Bachelor’s Degree	Xinjiang	Civil Servant
R28	Ms. Zhang	44	Master’s Degree	Xinjiang	University Faculty
R29	Mr. Li	52	Bachelor’s Degree	Xinjiang	Company Manager
R30	Mr. Wei	22	Junior high school and below	Xinjiang	Logistician

Appendix C The formal scale of URSCC

1-1	我认为城市安全的发展对我很重要<br>I think the development of urban safety is important to me
1-2	我觉得给他人普及公共安全方面的知识是积极有意义的<br>I think it is positive and meaningful to popularize the knowledge of public safety to others
1-3	在日常生活中遇到一些危害公共安全的行为，我觉得有义务去制止<br>I feel obliged to stop acts that endanger public safety in my daily life
2-1	我曾识别出某些自然灾害风险源（台风、高温、雾霾、地震等）<br>I have identified certain sources of natural disaster risk (typhoons, heat, haze, earthquakes, etc.)
2-2	我知道雾霾主要组成成分及其危害性<br>I know the main components of haze and its harmful effects
2-3	我参加过针对事故灾难危险源识别类的宣讲或培训<br>I have attended presentations or training on hazard identification for accidents and disasters
2-4	在使用易燃、易爆设备时，我会检查是否接地线<br>When using flammable or explosive equipment, I will check for an earth wire
2-5	我曾经识别出某些公共卫生危险源（传染病疫情、食品问题等）<br>I have identified certain sources of public health risk (infectious disease outbreaks, food issues, etc.)
2-6	我了解细菌、病毒所引起的传染病的具体病兆<br>I understand the specific signs of infectious diseases caused by bacteria and viruses
2-7	海产品附着菌类可低温存活，我会使用沸水煮烫后食用<br>I would use boiling water to boil the seafood and eat it as the mushrooms can survive at low temperatures
2-8	我曾识别出身边某些社会安全事件危险源（歹徒、有攻击性的动物等）<br>I have identified some social security incident hazards around me (thugs, aggressive animals, etc.)
2-9	我会避开照明度较差且行人较少的路段出行<br>I will avoid roads with poor lighting and fewer pedestrians
3-1	我能够预判出各类自然灾害的破坏程度<br>I can predict the extent of damage from various natural disasters
3-2	我知晓各个环境灾害预警级别所对应的破坏程度<br>I know the level of damage corresponding to each environmental hazard warning level
3-3	我会警惕周边可能发生的各类事故<br>I will be alert to all types of accidents that may occur in the vicinity
3-4	我可以预判出发生混乱时容易造成踩踏伤亡的公共区域<br>I can predict the public areas that are likely to cause a stampede in the event of chaos
3-5	当我乘坐公共交通工具时，我会注意其他乘客的情绪稳定性<br>When I travel on public transport, I am aware of the emotional stability of other passengers
3-6	我经历过遭遇公共卫生事件时（疫情、食物中毒等）的症状<br>I have experienced symptoms in the event of a public health incident (epidemic, food poisoning, etc.)
3-7	我会留意包装食品是否印有保质期及食品生产许可证<br>I will look out for packaged food with shelf life and food production license
3-8	我对不法分子（扒手、劫匪等）的作案手法有所了解<br>I have an understanding of criminal methods (pickpockets, robbers, etc.)
3-9	出行时我会注意是否有陌生人刻意接近<br>I look out for strangers approaching me when I travel

4-1	我应对过不同类型的自然灾害（台风、高温、暴雨、地震等）<br>I have dealt with different types of natural disasters (typhoons, heat, rainstorms, earthquakes, etc.)
4-2	台风过境时，我会第一时间加固门窗并检查电路<br>When a typhoon passes, I will be the first to reinforce doors and windows and check electrical circuits
4-3	我知悉我所居住城市的应急避难场所<br>I know the emergency shelters in the city I live in
4-4	我接受过某些突发事故（生产、交通、公共设施事故等）处理的讲座及培训<br>I have received lectures and training on the handling of unexpected accidents (production, traffic, utility accidents, etc.)
4-5	我能正确的使用灭火器（先拔出保险销，再压合压把，对准火焰根部喷射）<br>I can use a fire extinguisher correctly (remove the safety pin, then press the lever and aim the spray at the root of the fire)
4-6	我紧急处理过身边发生的公共卫生事件（传染病、食物中毒）<br>I have dealt with emergency public health incidents around me (infectious diseases, food poisoning)
4-7	我知道预防传染病的关键在于控制已感染人群、切断传播途径及保护易感人群<br>I know that the key to preventing infectious diseases is to control the infected population, cut off the transmission routes and protect the susceptible population
4-8	我经历过影响到我人身安全的事件，并采取了恰当的措施保证了自身安全<br>I have experienced incidents that have affected my personal safety and have taken appropriate measures to ensure my safety
4-9	当我发现被陌生人跟踪时，我会保持镇定并向商店、居民区等人多地带转移<br>When I notice that I am being followed by a stranger, I stay calm and move to a crowded area such as a shop or a residential area
5-1	周围人遇到危险时采取的安应对措施往往是因为我是这样做的<br>The safe response of those around me in danger is often because I do
5-2	我总能强烈影响我的亲人和朋友，使他们能够正确应对一些危险事件<br>I have always been able to strongly influence my family and friends so that they can respond properly to some dangerous events
5-3	在发生公共突发事件时，我能指挥和协调其他人进行应对<br>In the event of a public emergency, I can direct and coordinate the response of others
